# Supplementary material for: Plasmodium-infected erythrocytes induce secretion of IGFBP7 to form type II rosettes and escape phagocytosis
Source: eLife. 2020 Feb 18;9:e51546. doi: 10.7554/eLife.51546 (PMC7048393; doi:10.7554/eLife.51546)
Supplement: Figure 1—source data 1. — R = biological replicate (same parasite line, but different flasks of cultures, using the same URBCs and culture media). [file elife-51546-fig1-data1.docx]

**Figure 1- Source Data 1: Raw data (rosetting rates, %) for the data set presented in bar graph (1D).** R = biological replicate (same parasite line, but different flasks of cultures, using the same URBCs and culture media).

| *P. falciparum* lines | Control | | | CD14^+^ monocytes I | | | CD14^+^ monocytes II | | | CD14^-^ PBMCs I | | | CD14^-^ PBMCs II | | | THP-1 | | |
| --- | --- | --- | --- | --- | --- | --- | --- | --- | --- | --- | --- | --- | --- | --- | --- | --- | --- | --- |
|  | R1 | R2 | R3 | R1 | R2 | R3 | R1 | R2 | R3 | R1 | R2 | R3 | R1 | R2 | R3 | R1 | R2 | R3 |
| FVT402 | 5.0 | 5.0 | 6.0 | 15.0 | 17.0 | 18.0 | 14.0 | 16.0 | 17.0 | 6.0 | 6.0 | 5.0 | 5.0 | 6.0 | 5.0 | 18.0 | 17.0 | 15.0 |
| 3D7 | 3.0 | 4.0 | 2.0 | 7.0 | 8.0 | 7.0 | 7.0 | 9.0 | 9.0 | 3.0 | 3.0 | 4.0 | 3.5 | 4.0 | 3.0 | 8.0 | 7.0 | 7.0 |
| MKK183 | 2.0 | 2.0 | 2.0 | 6.0 | 6.0 | 7.0 | 7.0 | 8.0 | 6.0 | 2.0 | 3.0 | 3.0 | 2.0 | 3.0 | 2.0 | 6.5 | 7.0 | 6.0 |
