# Supplementary material for: Plasmodium-infected erythrocytes induce secretion of IGFBP7 to form type II rosettes and escape phagocytosis
Source: eLife. 2020 Feb 18;9:e51546. doi: 10.7554/eLife.51546 (PMC7048393; doi:10.7554/eLife.51546)
Supplement: Figure 5—source data 1. — R = biological replicate (same parasite line, but different flasks of cultures using the same URBCs and culture media). [file elife-51546-fig5-data1.docx]

**Figure 5- Source Data 1: Raw data (rosetting rates, %) for the data set presented in bar graph (5C).** R = biological replicate (same parasite line, but different flasks of cultures using the same URBCs and culture media).

| *P. falciparum* lines | NF54 VAR2CSA_WT | | NF54 T934D | |
| --- | --- | --- | --- | --- |
| R | Control | IGFBP7 | Control | IGFBP7 |
| 1 | 1.5 | 6.5 | 1.0 | 1.5 |
| 2 | 1.0 | 5.0 | 1.5 | 0.5 |
| 3 | 3.0 | 7.0 | 0.5 | 0.5 |
| 4 | 2.5 | 4.5 | 0.5 | 1.5 |
| 5 | 1.5 | 4.0 | 0.5 | 1.0 |
