# Supplementary material for: Plasmodium-infected erythrocytes induce secretion of IGFBP7 to form type II rosettes and escape phagocytosis
Source: eLife. 2020 Feb 18;9:e51546. doi: 10.7554/eLife.51546 (PMC7048393; doi:10.7554/eLife.51546)
Supplement: Figure 5—source data 2. — R = biological replicate (same lab-adapted parasite, but conducted on three cycles of ring stages from three different flasks of cultures using the same batch of URBCs and culture media). F = flask, C = cycle. [file elife-51546-fig5-data2.docx]

**Figure 5- Source Data 2: Raw data (rosetting rates, %) for the data set presented in bar graph (5D).** R = biological replicate (same lab-adapted parasite, but conducted on three cycles of ring stages from three different flasks of cultures using the same batch of URBCs and culture media). F = flask, C = cycle

| R (F/C) | IGFBP7-free | IGFBP7 100 ng/ml |
| --- | --- | --- |
| 1/1 | 3.0 | 6.0 |
| 2/1 | 4.5 | 8.5 |
| 3/1 | 4.0 | 7.0 |
| 1/2 | 3.0 | 8.0 |
| 2/2 | 2.5 | 6.0 |
| 3/2 | 1.5 | 7.0 |
| 1/3 | 2.0 | 8.0 |
| 2/3 | 1.0 | 5.0 |
| 3/3 | 1.0 | 6.0 |
