# Supplementary material for: Plasmodium-infected erythrocytes induce secretion of IGFBP7 to form type II rosettes and escape phagocytosis
Source: eLife. 2020 Feb 18;9:e51546. doi: 10.7554/eLife.51546 (PMC7048393; doi:10.7554/eLife.51546)
Supplement: Figure 8—source data 2. — R = biological replicate (same parasite, but different batches of cultures grown with different batches of URBCs). [file elife-51546-fig8-data2.docx]

**Figure 8- Source Data 2: Raw data (phagocytosis rates, %) for the data set presented in bar graph (8G).** R = biological replicate (same parasite, but different batches of cultures grown with different batches of URBCs).

| R | Monocytes  IGFBP7-free | Monocytes  IGFBP7 100 ng/ml | THP-1  IGFBP7-free | THP-1  IGFBP7 100 ng/ml |
| --- | --- | --- | --- | --- |
| 1 | 20.0 | 4.0 | 30.0 | 8.0 |
| 2 | 16.0 | 6.0 | 18.0 | 10.0 |
| 3 | 17.0 | 10.0 | 14.0 | 10.0 |
| 4 | 24.0 | 9.0 | 19.0 | 5.0 |
| 5 | 19.0 | 11.0 | 21.0 | 11.0 |
