# Supplementary material for: Plasmodium-infected erythrocytes induce secretion of IGFBP7 to form type II rosettes and escape phagocytosis
Source: eLife. 2020 Feb 18;9:e51546. doi: 10.7554/eLife.51546 (PMC7048393; doi:10.7554/eLife.51546)
Supplement: Supplementary file 1. [file elife-51546-supp1.docx]

| **Key Resources Table** | | | | |
| --- | --- | --- | --- | --- |
| **Reagent type (species) or resource** | **Designation** | **Source or reference** | **Identifiers** | **Additional information** |
| Biological sample (*Plasmodium falciparum*) | 3D7 | BEI Resources | MRA102 | Laboratory-adapted parasite strain; maintained in Singapore Immunology Network (SIgN) |
| Biological sample (*Plasmodium falciparum*) | FVT402 | This paper |  | Adapted from clinical isolate in Shoklo Malaria Research Unit (SMRU) |
| Biological sample (*Plasmodium falciparum*) | FVT201 | This paper |  | Adapted from clinical isolate in SMRU |
| Biological sample (*Plasmodium falciparum*) | MKK183 | This paper |  | Adapted from clinical isolate in SMRU |
| Biological sample (*Plasmodium falciparum*) | WPP3065 | This paper |  | Adapted from clinical isolate in SMRU |
| Biological sample (*Plasmodium falciparum*) | WPP2803 | This paper |  | Adapted from clinical isolate in SMRU |
| Biological sample (*Plasmodium falciparum*) | NHP1106 | This paper |  | Adapted from clinical isolate in SMRU |
| Biological sample (*Plasmodium falciparum*) | NHP4770 | This paper |  | Adapted from clinical isolate in SMRU |
| Biological sample (*Plasmodium falciparum*) | CS2-SBP1-KO | Maier et al.2007. doi:10.1182/blood-2006-08-043364  Chan et al.2016. doi:10.1007/s00018-016-2267-1. |  | Prepared in The Australian National University (ANU) and maintained in SIgN |
| Biological sample (*Plasmodium falciparum*) | CS2-WT | Maier et al.2007. doi:10.1182/blood-2006-08-043364  Chan et al.2016. doi:10.1007/s00018-016-2267-1. |  | Prepared in The Australian National University (ANU) and maintained in SIgN |
| Biological sample (*Plasmodium falciparum*) | NF54_VAR2CSA_WT | Dorin-Semblat et al.2019. doi: 10.1371/journal.pbio.3000308. |  | Provided by Benoit Gamain, Institut national de la santé et de la recherche médicale (INSERM) |
| Biological sample (*Plasmodium falciparum*) | NF54_T934D | Dorin-Semblat et al.2019. doi: 10.1371/journal.pbio.3000308 |  | Provided by Benoit Gamain, Institut national de la santé et de la recherche médicale (INSERM) |
| Cell line (Homo sapiens) | THP-1 | ATCC | TIB-202^™^ | Monocytic cell line |
| Other | Acridine Orange | ThermoFisher Scientific | Cat #1301 |  |
| Other | ÄKTA Micro System | GE Healthcare |  | Mass spectrometry |
| Chemical compound, drug | Albumax II | Gibco™ | Cat # 11021045 | For parasite culture |
| Antibody | Anti-A (ABO1 ) murine monoclonal antibody | TransClone® Bio-Rad | Cat # 86328 | Blood group-typing  (slide method: 50 µl) |
| Antibody | Anti-B (ABO2) murine monoclonal antibody | TransClone® Bio-Rad | Cat # 86470 | Blood group-typing  (slide method: 50 µl) |
| Other | BD Vacutainer™ with lithium heparin | ThermoFisher Scientific | Cat # 02-657-28 |  |
| Other | C-18 ReproSil Pur Basic beads 2.5 um | Dr Maisch | Cat #r125.b9 | Mass spectrometry |
| Other | CD14+ microbeads | Miltenyi Biotech | Cat # 130050201 |  |
| Other | Cellulose acetate syringe filter, pore size 0.45 µm | Sartorius Minisart®, Sigma-Aldrich | Cat # 16555-K |  |
| Other | Centrifuge | Sorvall® | Legend® RT Plus |  |
| Other | CF11 cellulose powder | Sigma-Aldrich® | Cat # 318094 |  |
| Other | EASY nLC1000 system | ThermoFisher Scientific | Cat #LC120 | Mass spectrometry |
| Other | Epifluorescence microscope | Nikon Eclipse | TS100 |  |
| Other | Falcon® Cell Culture Flask T25, filter cap | VWR™ | Cat #29185298 |  |
| Chemical compound, drug | Fetal Bovine Serum (FBS) | Gibco™ | Cat # 10500 | For cee line culture |
| Chemical compound, drug | Ficoll-paque | GE Healthcare | Cat # 17-5442-02 |  |
| Other | Flat bottom culture plate, 48 well | NUNC™ | Cat #150687 |  |
| Other | Flat bottom plate, 96 well | NUNC™ | Cat #44240421 |  |
| Other | Gemini 5u/C-18/110A, 150 mm x 1 mm column | Phenomenex | Cat #00F-4435-A0 | Mass spectrometry |
| Other | Giemsa | Merck | Cat # HX60416604 |  |
| Other | Glass coverslip 22x32mm | Mariendfeld | Cat # 0101112 |  |
| Software | GraphPad Prism7.0 | GraphPad |  | Statistical analysis |
| Chemical compound, drug | Heparinase I | R&D Systems® | Cat # 7897-GH-010 |  |
| Chemical compound, drug | Heparinase III | R&D Systems® | Cat # 6145-GH-010 |  |
| Chemical compound, drug | Hexadimethrine bromide | Sigma-Aldrich® | Cat # H9268 |  |
| Commercial assay or kit | Human IGFBP7 DuoSet® ELISA kit | R&D Systems® | Cat # DY009 |  |
| Commercial assay or kit | Human VWF SimpleStep ELISA® kit | Abcam | Cat # ab223864 |  |
| Chemical compound, drug | IMDM medium | Gibco™ | Cat #31980-030 |  |
| Other | Lab-Tek™ 8-chamber-slides | ThermoFisher Scientific | Cat #177445 |  |
| Other | Lysyl endopeptidase® (LysC) | Wako | Cat #125-02541 | Mass spectrometry |
| Other | MACS-LD columns | Miltenyi Biotec | Cat # 130042901 |  |
| Software | Mascot 2.5 | Matrix Science |  | Mass spectrometry |
| Chemical compound, drug | McCoy’s 5A medium | Gibco™ | Cat # 12330-031 |  |
| Other | Microplate reader Tecan i-Control | Tecan® |  |  |
| Commercial assay or kit | Mission™ shRNA Lentiviral transduction particles for knock down of IGFBP7 expression | Sigma-Aldrich® | hPGK-Puro_CMV-tGFP; SHCLNV-NM_001553; TRC#TRCN0000077943 |  |
| Commercial assay or kit | Mission™ shRNA Lentiviral transduction particles for knock down of Gly C expression | Sigma-Aldrich® | hPGK_CMV-tGFP; SHCLNV-NM_002101; TRC#TRCN0000437292 |  |
| Antibody | Mouse IgG, polyclonal-anti-human HABP2 | Abnova | Cat # H00003026-B01P | Working concentration: 25 µg/ml |
| Antibody | Mouse IgG, polyclonal-isotype control | Abcam | Cat # ab37355 | Working concentration: 25 µg/ml |
| Antibody | Mouse monoclonal IgG_1_, anti-human CFD | R&D Systems® | Cat # MAB18241 | Working concentration: 25 µg/ml |
| Antibody | Mouse monoclonal IgG_1_, anti-human CR1(CD35) | BD Pharmingen™ | Cat # 555451 | Working concentration: 25 µg/ml |
| Antibody | Mouse monoclonal IgG_1_, anti-human IGFBP7 | SinoBiological | Cat # 13100-MM01 | Working concentration: 25 µg/ml |
| Antibody | Mouse monoclonal IgG_1_, anti-human NID1 | R&D Systems® | Cat # MAB2570 | Working concentration: 25 µg/ml |
| Antibody | Mouse monoclonal IgG_1_, anti-human periostin/ OSF-2 | Sigma-Aldrich® | Cat # SAB4200197 | Working concentration: 25 µg/ml |
| Antibody | Mouse monoclonal IgG_1_ isotype control | R&D Systems® | Cat # MAB002 | Working concentration: 25 µg/ml |
| Antibody | Mouse monoclonal IgG_2B_, anti-human TSP-1 | R&D Systems® | Cat # MAB 3074 | Working concentration: 25 µg/ml |
| Antibody | Mouse monoclonal IgG_2B_ isotype control | R&D Systems® | Cat # MAB004 | Working concentration: 25 µg/ml |
| Commercial assay or kit | MycoAlert™ Plus Mycoplasma detection kit | Lonza | Cat # LT07-705 |  |
| Other | Orbitrap Fusion™ mass spectrometry | ThermoFisher Scientific | Cat # IQLAAEGAAPFADBMBCX | Mass spectrometry |
| Chemical compound, drug | Percoll® | Sigma-Aldrich® | Cat # P1644 |  |
| Software | Proteome Discoverer 1.4 software | ThermoFisher Scientific |  | Mass spectrometry |
| Chemical compound, drug | Puromycin | Sigma-Aldrich® | Cat # 9620 |  |
| Antibody | Rabbit IgG, polyclonal-anti-human VWF | Abcam | Cat # ab6994 | Working concentration: 25 µg/ml |
| Antibody | Rabbit IgG, polyclonal-isotype control | Abcam | Cat # ab37415 | Working concentration: 25 µg/ml |
| Recombinant protein | Recombinant human interferon gamma IFNγ | R&D Systems® | Cat #285-IF |  |
| Recombinant protein | Recombinant human IGFBP7 (rhIGFBP7) | ProSpec | Cat # cyt-788 |  |
| Recombinant protein | Recombinant human VWF (rhVWF) | Abcam | Cat # ab152801 |  |
| Chemical compound, drug | RPMI 1640 medium | HyClone™ | Cat # SH30255.01 |  |
| Other | Sep-Pak C-18 columns | Waters | Cat # WAT051910 | Mass spectrometry |
| Other | Trypan Blue | Sigma-Aldrich® | Cat #T6146 |  |
| Chemical compound, drug | Trypsin | HyClone™ | Cat # SV30031.01 |  |
| Chemical compound, drug | Trypsin Gold, Mass Spectrometry Grade | Promega | Cat #V5280 | Mass spectrometry |
| Other | Vivaspin20 twin PES membrane 30 kDa concentrator | Sartorius | Cat # Z629472 |  |
| Other | Zymosan A | Sigma-Aldrich® | Cat # Z4250 |  |
