# Supplementary material for: Plasmodium-infected erythrocytes induce secretion of IGFBP7 to form type II rosettes and escape phagocytosis
Source: eLife. 2020 Feb 18;9:e51546. doi: 10.7554/eLife.51546 (PMC7048393; doi:10.7554/eLife.51546)
Supplement: Supplementary file 3. [file elife-51546-supp3.docx]

**Supplementary file 3: Shortlisted candidates from a list of 694 compounds identified by mass spectrometry.**

| Name | Score  A (3,6) | Coverage  A (3,6) | #Peptides  A (3,6) | Σ# unique peptides | #PSM  A(3,6) |
| --- | --- | --- | --- | --- | --- |
| Insulin-like growth factor-binding protein 7 (IGFBP7) | 175.31 | 17.56 | 6 | 6 | 7 |
| Complement factor D (CFD) | 59.50 | 8.70 | 1 | 1 | 1 |
| Nidogen 1 (NID1) | 312.18 | 8.35 | 8 | 8 | 17 |
| Hyaluronan-binding protein 2 (HABP2) | 18.08 | 8.21 | 2 | 2 | 2 |
| Periostin (POSTN) | 65.82 | 5.32 | 5 | 1 | 8 |
