# Supplementary material for: Plasmodium-infected erythrocytes induce secretion of IGFBP7 to form type II rosettes and escape phagocytosis
Source: eLife. 2020 Feb 18;9:e51546. doi: 10.7554/eLife.51546 (PMC7048393; doi:10.7554/eLife.51546)
Supplement: Supplementary file 4. [file elife-51546-supp4.docx]

**Supplementary file 4. Recruited *P. falciparum* clinical isolates from the Thai-Burmese Border.**

| **Isolate** | **Gender** | **Age** | **Parasitemia, %** | **Blood group** | **Used for experiments** |
| --- | --- | --- | --- | --- | --- |
| PID408028 | M | 26 | 1 | B | 1B |
| RDM00036 | M | 63 | 3.4 | O | 1B |
| RDM00037 | F | 8 | 2.3 | O | 1B |
| RDM103 | M | 41 | 0.4 | B | 2A |
| PID118313 | M | 20 | 0.6 | B | 2A |
| PID403540 | F | 3 | 1 | B | 2A |
| TH004069 | M | 31 | 1 | AB | 2A, 2B, 3A, 3B, 3C |
| MRC0135 | M | 40 | 1.3 | A | 2A, 2B, 3A, 3B, 3C |
| NHP2188 | M | 23 | 19.2 | A | 2A, 2B, 3A, 3B, 3C, 3D, 3E |
| NHP1403 | F | 61 | 9.5 | O | 2A, 2B, 3A, 3B, 3C, 3D, 3E, 4A, 6D |
| NHP1483 | F | 17 | 4.2 | B | 2A, 2B, 3A, 3B, 3C, 3D, 3E, 4C, 4E |
| PID314265 | F | 41 | 0.9 | O | 2A, 2B, 3A, 3B, 3C, 3D, 3E, 4C, 4E |
| HP374 | F | 19 | 6.1 | A | 2A, 2B, 3A, 3B, 3C, 3D, 3E, 4C, 4E |
| PID104582 | F | 25 | 0.1 | O | 2A, 2B, 3A, 3B, 3C, 3E, 4C, 4E |
| OZ228 | M | 33 | 0.2 | O | 2B, 3A, 3B, 3C, 3E, 4A, 6A, 6B |
| PID403035 | M | 23 | 0.3 | B | 4A, 6A, 6B, 4C, 4E |
| RDM094 | M | 5 | 1 | B | 4A, 6A, 6B, 6F |
| MMA2156 | F | 58 | 0.7 | O | 4A, 6A, 6B, 6F |
| HP357 | F | 32 | 4.3 | A | 4A, 6A, 6B, 6F |
| OZ234 | M | N/A | 0.1 | O | 6A, 6B, 6F |
| NHP1454 | M | 35 | 4 | A | 4A, 6A, 6B, 6D, 6F |
| HP3069 | F | 17 | 6.3 | B | 6F, 6H |
| MMA4044 | M | 31 | 0.3 | B | 6F, 6H |
| PID423677 | M | 17 | 2.5 | AB | 5A, 6H |
| NHP1338 | M | 35 | 5.2 | AB | 5A, 6H |
| RDM104 | M | 19 | 0.5 | B | 5A, 6H |
| NHP4770 | M | 33 | 4.2 | O | 4A, 6D |
| NHP1481 | F | 4 | 4.4 | B | 4A, 6D |
| NHP0319 | M | 25 | 4.1 | O | 4A, 6D |
| NHP3048 | M | 13 | 6 | A | 4A, 6D |
| NHP4703 | F | 41 | 4.5 | AB | 6D |
| NHP1386 | M | 7 | 15 | A | 6D |
| NHP1401 | F | 19 | 4 | B | 6D, 5A |
| NHP3127 | F | 50 | 4.3 | B | 6D |
| NHP4265 | F | 7 | 8 | B | 6D, 5A |
