# Supplementary material for: Plasmodium-infected erythrocytes induce secretion of IGFBP7 to form type II rosettes and escape phagocytosis
Source: eLife. 2020 Feb 18;9:e51546. doi: 10.7554/eLife.51546 (PMC7048393; doi:10.7554/eLife.51546)
Supplement: Supplementary file 5. [file elife-51546-supp5.docx]

**Supplementary file 5. Recruited *P. vivax* clinical isolates from the Thai-Burmese Border.**

| **Isolate** | **Gender** | **Age** | **Parasitemia, %** | **Blood group** | **Used for experiments** |
| --- | --- | --- | --- | --- | --- |
| BPD 0483 | M | 45 | 0.7 | O | 1B |
| PID411161 | F | 22 | 0.6 | O | 1B |
| PID406704 | M | 33 | 0.6 | B | 1B |
| PID112410 | F | 11 | 0.4 | A | 1B |
| PID416460 | F | 63 | 0.3 | O | 1B |
| PID403915 | F | 41 | 0.6 | O | 1B |
| PID112038 | M | 14 | 0.3 | O | 1B |
| PID416510 | M | 21 | 0.5 | B | 1B |
| BPD491 | M | 10 | 0.3 | O | 1B |
| BPD493 | M | 59 | 0.9 | O | 1B |
| PID403130 | M | 17 | 0.3 | B | 1B |
| PID416546 | M | 21 | 0.4 | A | 1B |
| BPD494 | F | 41 | 0.4 | B | 1B |
| PID100197 | M | 28 | 0.2 | B | 1B |
| PID314183 | M | 16 | 0.03 | O | 2A |
| PID422801 | M | 50 | 0.2 | O | 2A |
| THV004082 | F | 54 | 0.1 | A | 2A |
| PID418099 | M | 17 | 0.03 | O | 2A |
| THV004084 | M | 53 | 0.03 | B | 2A |
| THV004083 | M | 18 | 0.02 | B | 2A |
| PID404586 | F | 6 | 0.4 | B | 2A, 6A, 6B |
| THV004039 | F | 43 | 0.02 | O | 2A |
| PID313030 | F | 57 | 0.3 | O | 2A |
| PID422901 | M | 43 | 0.3 | B | 6A, 6B |
| PID406998 | F | 4 | 0.3 | O | 2C |
| PID409065 | F | 6 | 0.4 | A | 2C, 3A |
| PID424107 | M | 16 | 0.3 | A | 2C, 3A, 3B, 3C, 3D, 3E, 6A, 6B |
| PID416055 | F | 6 | 0.5 | B | 2C, 3A, 3B, 3C, 3D, 3E |
| PID116351 | F | 24 | 0.4 | O | 2C, 3A, 3B, 3C, 3D, 3E, 6A, 6B |
| PID424121 | M | 6 | 0.05 | B | 2C, 3A, 3B, 3C, 3D, 3E |
| PID109874 | F | 28 | 0.03 | O | 2C, 3A, 3B, 3C, 3D, 3E |
| PID423369 | M | 30 | 0.4 | B | 2C, 3A, 3B, 3C, 3D, 3E |
| PID406998 | F | 6 | 0.7 | O | 2C, 3A, 3B, 3C, 3D |
| PID403063 | M | 17 | 0.3 | B | 2C, 3A, 3B, 3C, 3D |
| PID108989 | M | 29 | 0.05 | O | 2C, 3A, 3B, 3C, 3D, 6A, 6B |
| PID423135 | F | 14 | 0.4 | O | 2C, 3A, 3B, 3C, 3D |
| PID418481 | M | 8 | 0.03 | AB | 2C, 3A, 3B, 3D, 4D |
| PID404330 | F | 10 | 0.1 | AB | 2C, 3A, 3B, 3C, 3D, 4D |
| PID409345 | M | 13 | 0.7 | O | 2C, 3A, 3B, 3C, 3D, 4D, 6A, 6B |
| PID423814 | M | 10 | 0.3 | O | 2C, 3A, 3B, 3D, 4D |
| PID111380 | M | 45 | 0.05 | A | 2C, 3A, 3B, 3D, 4D |
| PID423764 | M | 45 | 0.3 | O | 2C, 3A, 3B, 3D, 4D |
| PID105805 | M | 12 | 0.3 | A | 2C, 3A, 3B, 3D, 4D |
| PID412151 | F | 57 | 0.5 | O | 2C, 3A, 3B, 3D, 4D |
| PID415994 | M | 2 | 0.5 | O | 2C, 3A, 3B, 3C, 3D, 4D, 6A, 6B |
| PID414711 | F | 8 | 0.3 | B | 2C, 3A, 3B, 3D, 4D |
| PID424477 | M | 4 | 0.3 | O | 3A, 3B, 3D, 4D, 6A, 6B |
| PID422693 | M | 38 | 0.7 | O | 6A, 6B |
| PID424506 | F | 13 | 0.05 | O | 6A, 6B |
| PID406889 | F | 18 | 0.1 | AB | 6A, 6B |
| PID401131 | F | 13 | 1.1 | A | 4F, 5A, 6I |
| PID425407 | M | 27 | 0.03 | O | 4F, 5A |
| PID425544 | M | 13 | 0.3 | O | 4F, 5A, 6I |
| PID302114 | F | 20 | 0.5 | A | 4F, 5A |
| PID109371 | F | 15 | 0.2 | O | 6G |
| PID108371 | M | 22 | 0.2 | B | 6G |
| PID314653 | M | 20 | 0.8 | B | 6G, 6I |
| PID421209 | M | 4 | 0.5 | O | 6G, 6I |
| PID419197 | F | 2 | 0.5 | O | 6G, 6I |
| THV004096 | F | 47 | 0.5 | O | 6G |
| PID410117 | F | 6 | 0.8 | O | 6G |
| PID406998 | F | 6 | 0.7 | O | 6I |
| PID424601 | F | 5 | 2 | B | 6I |
| BPD472 | F | N/A | 0.5 | A | 6I |
| PID425305 | M | 13 | 0.03 | O | 6I |
| PID427120 | F | 9 | 0.3 | A | 6E |
| PID323623 | F | 12 | 0.3 | B | 6E |
| PID121917 | M | 15 | 0.1 | O | 6E |
| PID122557 | M | 17 | 0.2 | O | 6E |
| PID427316 | M | 51 | 0.1 | B | 6E |
| PID428734 | M | 24 | 0.9 | AB | 4B |
| DMA520 | F | 26 | 0.1 | B | 4B |
| PID427309 | F | 14 | 2.8 | B | 4B |
| PID122775 | N/A | 19 | 0.4 | O | 4B |
| PID428843 | M | 23 | 0.1 | B | 4B |
| PID423539 | M | 14 | 0.5 | A | 4B |
