# Supplementary material for: Plasmodium-infected erythrocytes induce secretion of IGFBP7 to form type II rosettes and escape phagocytosis
Source: eLife. 2020 Feb 18;9:e51546. doi: 10.7554/eLife.51546 (PMC7048393; doi:10.7554/eLife.51546)
Supplement: Supplementary file 6. — Flow chart showing the experiments done in the project, along with the number of samples recruited for each experiment. [file elife-51546-supp6.docx]

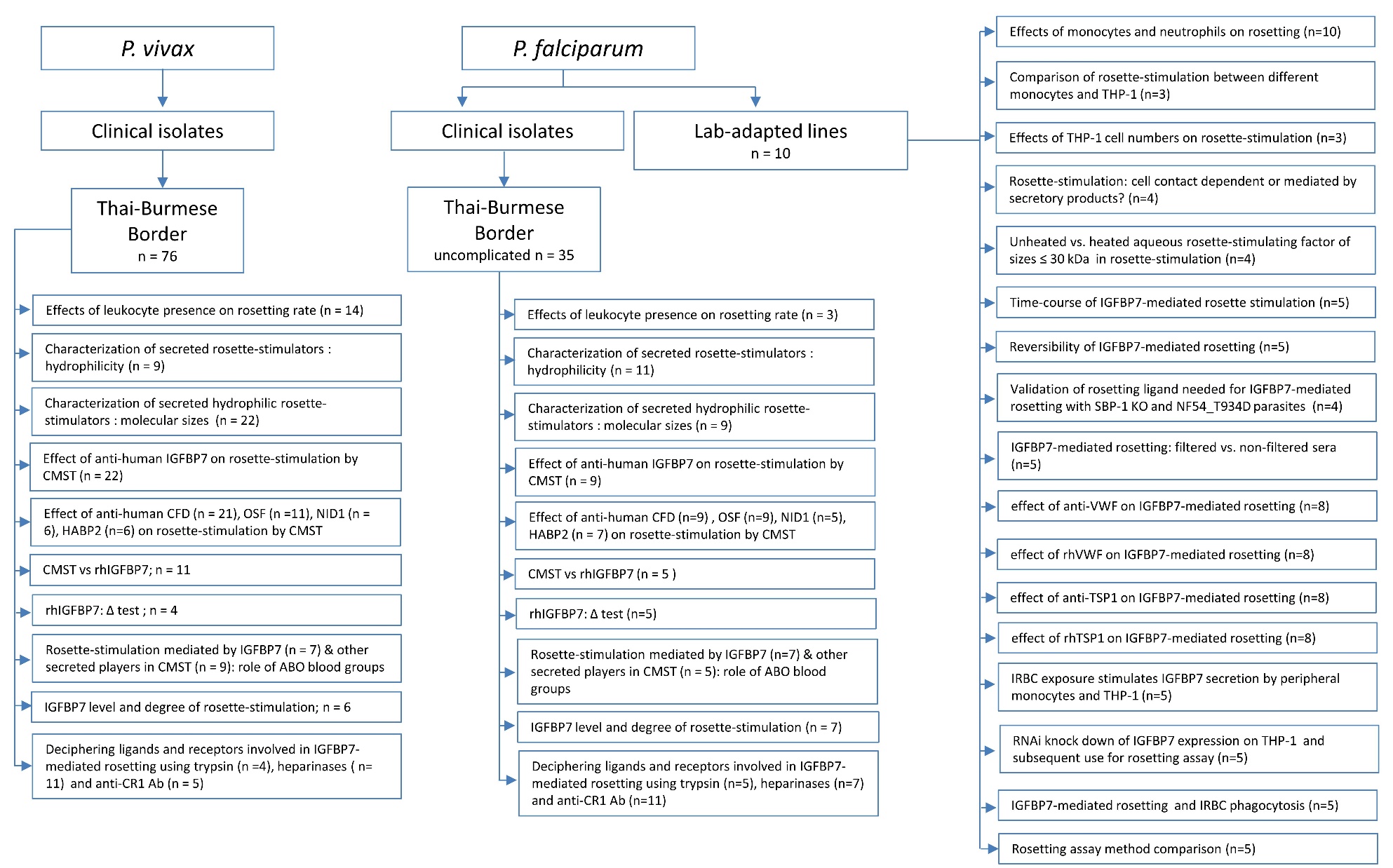


**Supplementary file 6: Experiment flow.** Flow chart showing the experiments done in the project, along with the number of samples recruited for each experiment.
