# Supplementary material for: Plasmodium-infected erythrocytes induce secretion of IGFBP7 to form type II rosettes and escape phagocytosis
Source: eLife. 2020 Feb 18;9:e51546. doi: 10.7554/eLife.51546 (PMC7048393; doi:10.7554/eLife.51546)
Supplement: Supplementary file 7. — (A) Plot of rosetting rates obtained from recruited P. falciparum lines (n = 5) using different wet mount methods, with insets underneath the x-axis showing rosettes visualized by respective methods [immersion oil (1000×) magnification, scale bars represent 10 µm]. Pictures of unstained and Giemsa-wet mounts were taken using a light microscope Olympus BX43, whereas pictures of the acridine orange-wet mount were taken on an epifluorescence microscope Nikon TS100. One-way ANOVA with Tukey’s test: unstained vs. Giemsa: p=0.9517. Acridine orange vs. unstained p>0.9999. Acridine orange vs. Giemsa: p=0.9809. (B) Changes of rosetting rates by IGFBP7 collected using different rosetting assays. Dotted lines were used to show read ups collected from different methods on the same sample. Dataset Giemsa did not pass normality test (Shapiro-Wilk normality test). Friedman with Dunn’s test: unstained vs. Giemsa: p=0.3415; unstained vs. acridine orange: p=0.6177; Giemsa vs. acridine orange: p>0.9999, that is there was no significant difference between the methods used. n.s. not significant. [file elife-51546-supp7.docx]

**
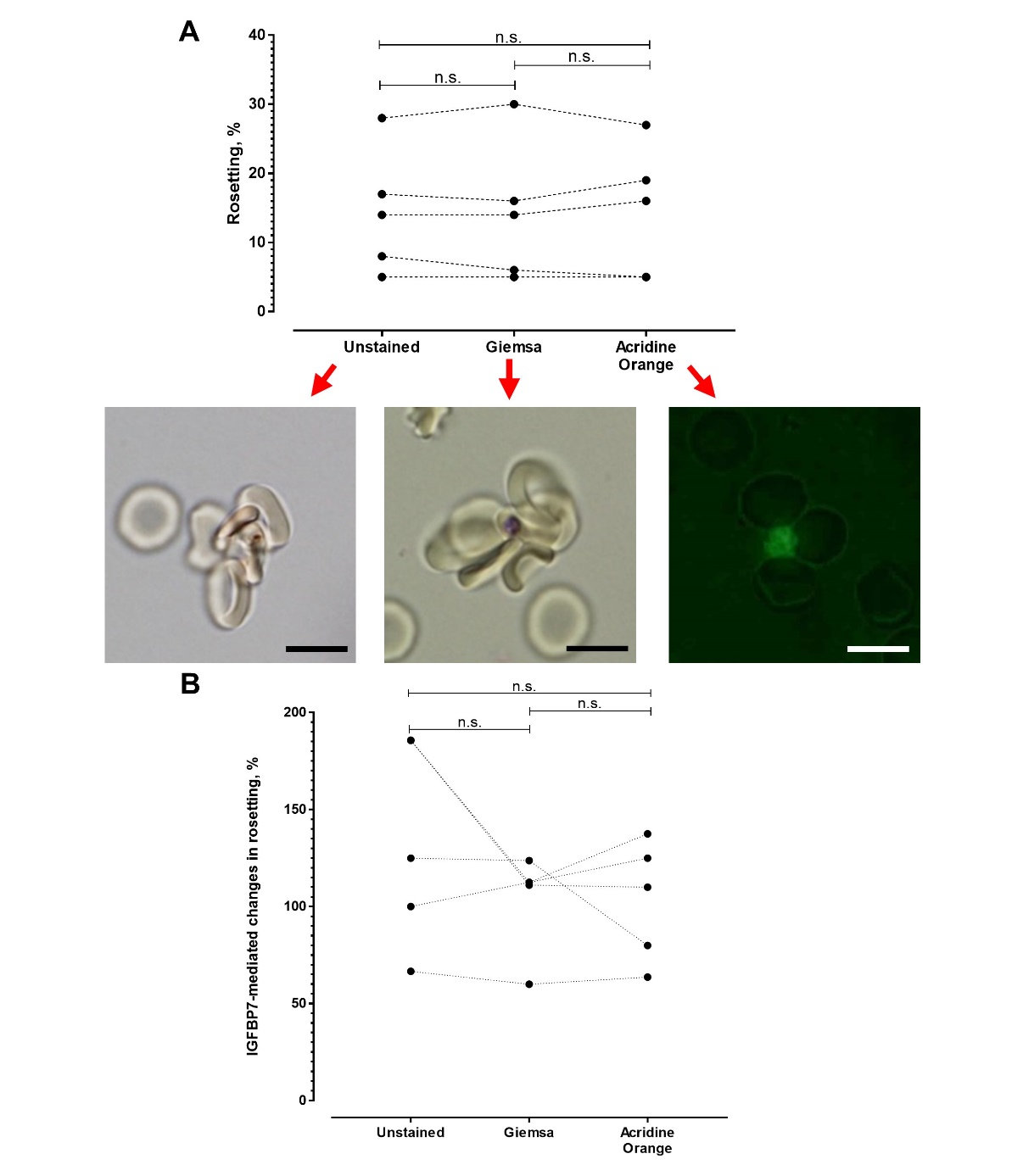
**

**Supplementary file 7. Method comparison for rosetting assay. (A)** Plot of rosetting rates obtained from recruited *P. falciparum* lines (n = 5) using different wet mount methods, with insets underneath the x-axis showing rosettes visualized by respective methods [immersion oil (1000X) magnification, scale bars represent 10µm]. Pictures of unstained and Giemsa-wet mounts were taken on light microscope Olympus BX43 whereas picture of acridine orange-wet mount was taken on epifluorescence microscope Nikon TS100. One-way ANOVA with Tukey’s test: unstained vs. Giemsa: P = 0.9517. Acridine orange vs. unstained P > 0.9999. Acridine orange vs. Giemsa: P = 0.9809. **(B)** Changes of rosetting rates by IGFBP7 collected using different rosetting assays. Dotted lines were used to show read ups collected from different methods on the same sample. Dataset Giemsa did not pass normality test (Shapiro-Wilk normality test). Friedman with Dunn’s test: unstained vs. Giemsa: P = 0.3415; unstained vs. acridine orange: P = 0.6177; Giemsa vs. acridine orange: P > 0.9999. i.e. no significant difference between the methods used. n.s. not significant.
