# Supplementary material for: Plasmodium-infected erythrocytes induce secretion of IGFBP7 to form type II rosettes and escape phagocytosis
Source: eLife. 2020 Feb 18;9:e51546. doi: 10.7554/eLife.51546 (PMC7048393; doi:10.7554/eLife.51546)
Supplement: Supplementary file 9. [file elife-51546-supp9.docx]

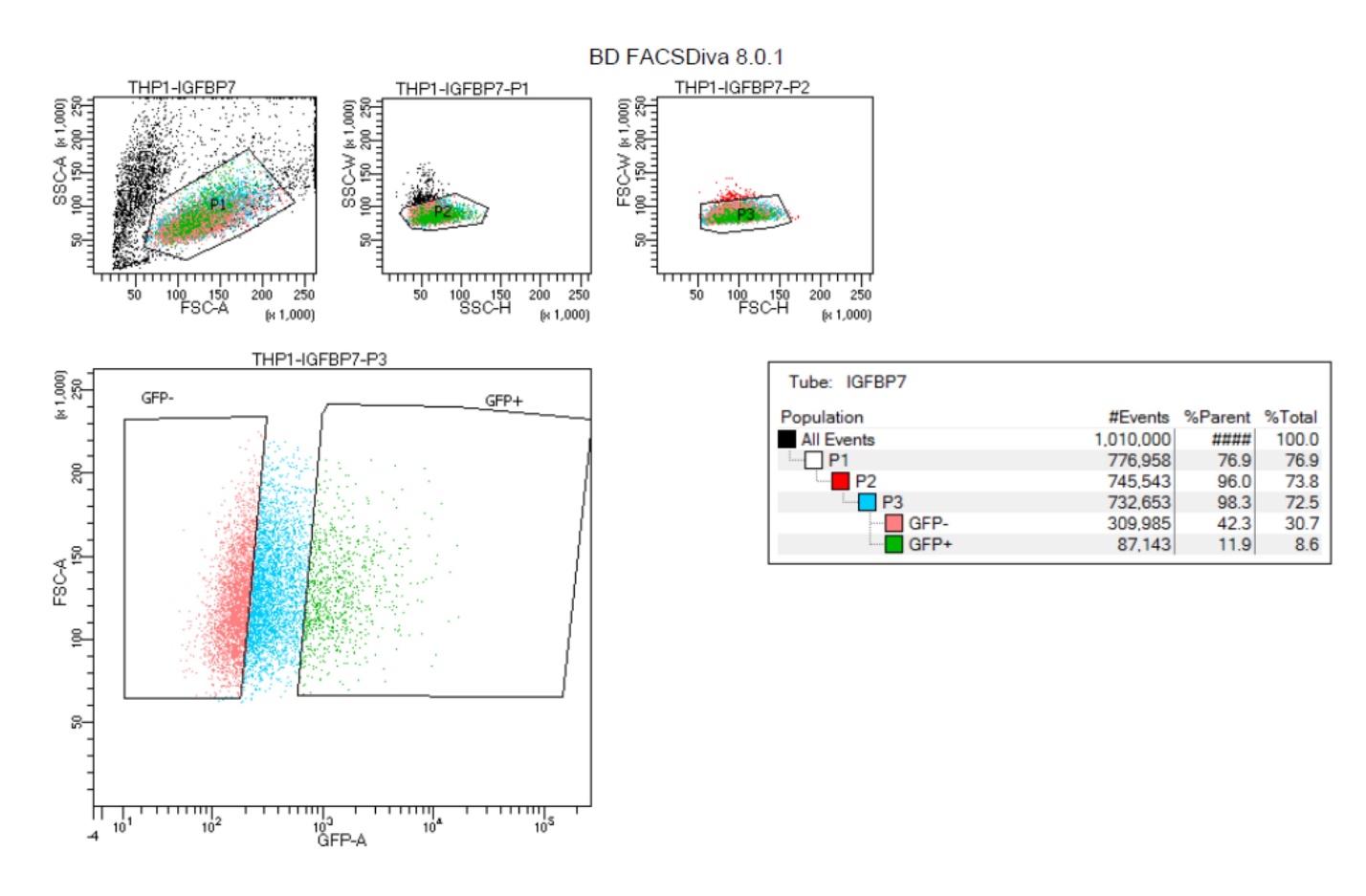


**Supplementary file 9. Profiling of IGFBP-KD THP-1 cell population prior to green fluorescent protein (GFP)-based sorting post-shRNA transduction.**
