# Supplementary material for: Plasmodium-infected erythrocytes induce secretion of IGFBP7 to form type II rosettes and escape phagocytosis
Source: eLife. 2020 Feb 18;9:e51546. doi: 10.7554/eLife.51546 (PMC7048393; doi:10.7554/eLife.51546)
Supplement: Supplementary file 10. [file elife-51546-supp10.docx]

**
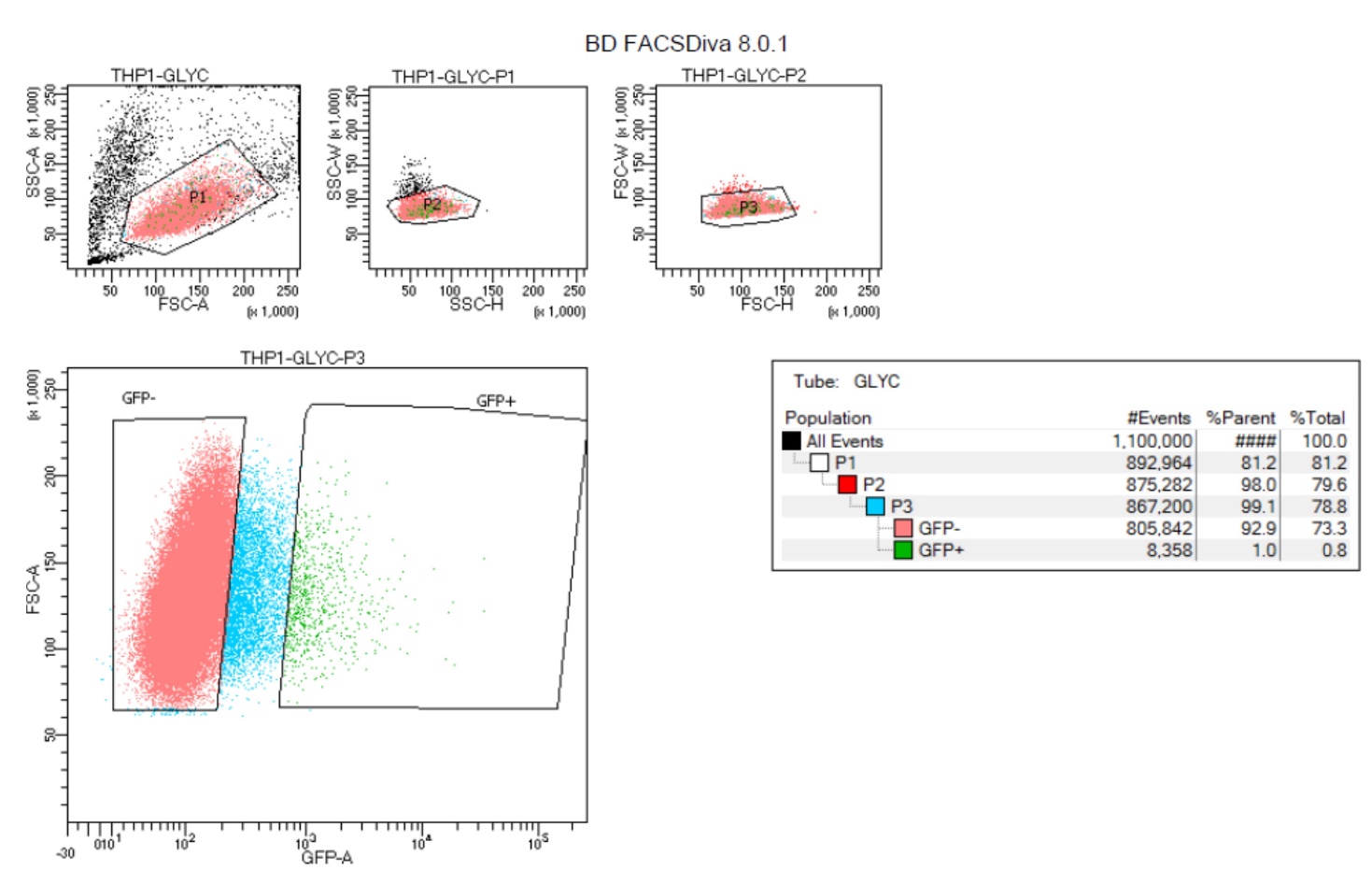
**

**Supplementary file 10. Profiling of GlyC-KD THP-1 cell population prior to green fluorescent protein (GFP)-based sorting post-shRNA transduction.**
