# Supplementary material for: Plasmodium-infected erythrocytes induce secretion of IGFBP7 to form type II rosettes and escape phagocytosis
Source: eLife. 2020 Feb 18;9:e51546. doi: 10.7554/eLife.51546 (PMC7048393; doi:10.7554/eLife.51546)
Supplement: Transparent reporting form [file elife-51546-transrepform.docx]

***eLife’s* transparent reporting form**

We encourage authors to provide detailed information *within their submission* to facilitate the interpretation and replication of experiments. Authors can upload supporting documentation to indicate the use of appropriate reporting guidelines for health-related research (see [EQUATOR Network](http://www.equator-network.org/%20)), life science research (see the [BioSharing Information Resource](https://biosharing.org/)), or the [ARRIVE guidelines](http://www.plosbiology.org/article/info:doi/10.1371/journal.pbio.1000412) for reporting work involving animal research. Where applicable, authors should refer to any relevant reporting standards documents in this form.

If you have any questions, please consult our Journal Policies and/or contact us: [editorial@elifesciences.org](mailto:editorial@elifesciences.org).

**Sample-size estimation**

- You should state whether an appropriate sample size was computed when the study was being designed
- You should state the statistical method of sample size computation and any required assumptions
- If no explicit power analysis was used, you should describe how you decided what sample (replicate) size (number) to use

Please outline where this information can be found within the submission (e.g., sections or figure legends), or explain why this information doesn’t apply to your submission:

We did not use explicit power analysis in the study design. Nevertheless, a total of 35 *P. falciparum* clinical isolates, 76 *P. vivax* clinical isolates and 12 laboratory-adapted *P. falciparum* lines were used throughout this study. The information is available in Supplementary files 1, 4, 5. Due to the difficulties to adapt clinical isolates to sustainable *in vitro* cultivation conditions, the n numbers of each experiment differ. Normality test was conducted prior to analysis, as stated in the method (statistical analysis) and the n number for each experiment is stated in figure legend.

**Replicates**

- You should report how often each experiment was performed
- You should include a definition of biological versus technical replication
- The data obtained should be provided and sufficient information should be provided to indicate the number of independent biological and/or technical replicates
- If you encountered any outliers, you should describe how these were handled
- Criteria for exclusion/inclusion of data should be clearly stated
- High-throughput sequence data should be uploaded before submission, with a private link for reviewers provided (these are available from both GEO and ArrayExpress)

Please outline where this information can be found within the submission (e.g., sections or figure legends), or explain why this information doesn’t apply to your submission:

We have defined “biological replicates” in the methods. We have stated the replicate number clearly in the figure legends and methods.

Data of all the biological replicates (different parasite lines) are described or explained in the figure legends and in Supplementary files 1, 4, 5.

**Statistical reporting**

- Statistical analysis methods should be described and justified
- Raw data should be presented in figures whenever informative to do so (typically when N per group is less than 10)
- For each experiment, you should identify the statistical tests used, exact values of N, definitions of center, methods of multiple test correction, and dispersion and precision measures (e.g., mean, median, SD, SEM, confidence intervals; and, for the major substantive results, a measure of effect size (e.g., Pearson's r, Cohen's d)
- Report exact p-values wherever possible alongside the summary statistics and 95% confidence intervals. These should be reported for all key questions and not only when the p-value is less than 0.05.

The type of tests used was decided after running normality test, as stated in the methods. The type of test used for each set of data, the p values, and n number are stated in the figure legends. For the only large data file (Supplementary file 2) in this manuscript, which is the data generated from the mass spectrometry, the decision was made based on the scores generated by the mass spectrometry (available in Supplementary file2), along with other criteria such as the reported cellular functions of the candidates, as stated in the manuscript. The softwares and steps used for the analysis and interpretation of this dataset are stated in the Methods, and in the key resources table (Supplementary file 1_Key Resources Table).

Please outline where this information can be found within the submission (e.g., sections or figure legends), or explain why this information doesn’t apply to your submission:

(For large datasets, or papers with a very large number of statistical tests, you may upload a single table file with tests, Ns, etc., with reference to sections in the manuscript.)

**Group allocation**

- Indicate how samples were allocated into experimental groups (in the case of clinical studies, please specify allocation to treatment method); if randomization was used, please also state if restricted randomization was applied
- Indicate if masking was used during group allocation, data collection and/or data analysis

Please outline where this information can be found within the submission (e.g., sections or figure legends), or explain why this information doesn’t apply to your submission:

Upon arrival at our laboratory premise, the samples were used in both treated and untreated conditions tested (to attain paired comparisons). A workflow of study is prepared (supplementary file 6) and the procedure is stated in methods section.

**Additional data files (“source data”)**

- We encourage you to upload relevant additional data files, such as numerical data that are represented as a graph in a figure, or as a summary table
- Where provided, these should be in the most useful format, and they can be uploaded as “Source data” files linked to a main figure or table
- Include model definition files including the full list of parameters used
- Include code used for data analysis (e.g., R, MatLab)
- Avoid stating that data files are “available upon request”

Please indicate the figures or tables for which source data files have been provided:

Data represented as bar graphs are provided as source data tables (5 sets): Figure 1-Source data 1; Figure 5-Source data 1; Figure5-Source data 2; Figure 8- Source data 1; Figure 8- Source data 2
